# Rapid Holocene thinning of an East Antarctic outlet glacier driven by marine ice sheet instability

**DOI:** 10.1038/ncomms9910

**Published:** 2015-11-26

**Authors:** R. S. Jones, A. N. Mackintosh, K. P. Norton, N. R. Golledge, C. J. Fogwill, P. W. Kubik, M. Christl, S. L. Greenwood

**Affiliations:** 1 Antarctic Research Centre, Victoria University of Wellington, Kelburn, Wellington 6012, New Zealand; 2 School of Geography, Environment and Earth Sciences, Victoria University of Wellington, Kelburn, Wellington 6012, New Zealand; 3 GNS Science, Avalon, Lower Hutt 5011, New Zealand; 4 Climate Change Research Centre, University of New South Wales, Sydney 2052, Australia; 5 Laboratory of Ion Beam Physics, Department of Physics, ETH Zurich, Otto Stern Weg 5, 8093 Zurich, Switzerland; 6 Department of Geological Sciences, Stockholm University, 10691 Stockholm, Sweden

## Abstract

Outlet glaciers grounded on a bed that deepens inland and extends below sea level are potentially vulnerable to ‘marine ice sheet instability'. This instability, which may lead to runaway ice loss, has been simulated in models, but its consequences have not been directly observed in geological records. Here we provide new surface-exposure ages from an outlet of the East Antarctic Ice Sheet that reveal rapid glacier thinning occurred approximately 7,000 years ago, in the absence of large environmental changes. Glacier thinning persisted for more than two and a half centuries, resulting in hundreds of metres of ice loss. Numerical simulations indicate that ice surface drawdown accelerated when the otherwise steadily retreating glacier encountered a bedrock trough. Together, the geological reconstruction and numerical simulations suggest that centennial-scale glacier thinning arose from unstable grounding line retreat. Capturing these instability processes in ice sheet models is important for predicting Antarctica's future contribution to sea level change.

## Abstract (web summary)

Irreversible ice loss from East Antarctic outlet glaciers during periods of ice sheet instability is yet to be observed in the geological record. Here, Jones *et al*. combine surface-exposure ages and model simulations to show the centennial-scale glacier thinning of Mackay Glacier during the mid-Holocene.

## Graphical abstract

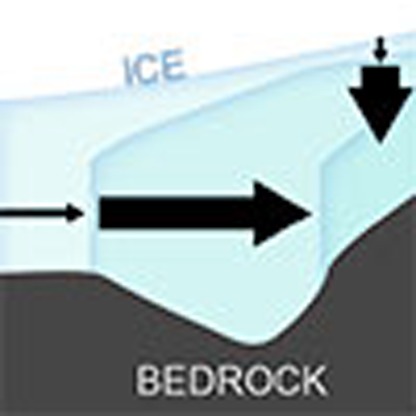

The East Antarctic Ice Sheet (EAIS) contains more than two-thirds of the total volume of Antarctic ice grounded below sea level^1^, which is potentially vulnerable to marine ice sheet instability^2^,^3^,^4^,^5^,^6^. This instability occurs when initial grounding line retreat into deeper water leads to thicker ice at the grounding line that is closer to floatation, causing increased ice flux and glacier thinning, a positive feedback that results in further grounding line retreat and drawdown of ice^5^,^7^,^8^. Satellite observations suggest that parts of the EAIS are currently experiencing dynamic thinning, and outlet glacier retreat^9^,^10^. This thinning is most likely initiated at the bed and terminus^11^,^12^, and may ultimately lead to irreversible mass loss if local topographic conditions favour retreat beyond a stability threshold^5^,^6^. Current understanding of self-sustaining glacier retreat is based on the short-lived tidewater cycles of marine-terminating glaciers in temperate and sub-polar settings^7^,^8^,^13^,^14^. Geological data can be used to extend the record of ice sheet observations in Antarctica, providing rates, durations and magnitudes of ice surface lowering that may have resulted from instability processes in the period preceding the satellite era.

Here we report a new terrestrial record of ice sheet thinning from Mackay Glacier, an outlet of the EAIS (Fig. 1). Mackay Glacier is suitably located to investigate the effects of marine ice sheet instability because: (1) a tectonically and glacially formed overdeepening occurs immediately offshore of the glacier^15^; (2) Mackay Glacier is laterally confined, and can be simulated using a one-dimensional flowline model; and (3) a relatively high concentration of offshore and onshore chronologies provide regional context, recording when Mackay Glacier became independent from grounded ice in the western Ross Sea during the last deglaciation^16^. Our record identifies a period of abrupt glacier change during the mid-Holocene. Using empirical data to constrain a high-resolution numerical model, we link changes in glacier surface elevation to the most likely driver of ice sheet retreat. In our model simulations, retreat of the grounding line across a landward-deepening bed is accompanied onshore by centennial-scale glacier thinning of the same magnitude as that shown by the terrestrial surface-exposure dates. On this basis, we argue that our surface-exposure data provide direct evidence of rapid topographically induced ice sheet retreat in Antarctica.

## Results

### Chronology of ice surface lowering

Dating of glacial erratic clasts in altitudinal transects has the potential to record the past surface lowering of a glacier from a previously thicker configuration^17^. We targeted two nunataks in the lower reaches of Mackay Glacier to determine its thinning history from the Last Glacial Maximum (LGM) to the present day. Mt Suess/Gondola Ridge lies adjacent to the main flow path of Mackay Glacier, ∼7–14-km upstream from the present-day grounding line and 15–25 km from the terminus. Present-day ice flow reaches velocities of 180 m per year^18^ alongside this exposed basaltic and granitic bedrock. The steep, ice-abraded flanks of Mt Suess and glacially smoothed and streamlined topography of Gondola Ridge provide evidence of thicker ice in the past. Sandstone and quartzite glacial erratics of the Beacon Supergroup are found resting on both weathered and striated bedrock, and extend up to 890 m above sea level (m a.s.l.) on Mt Suess (∼340 m above the proximal modern ice surface). Low Ridge presently flanks the glacier terminus, 1–4.5 km downstream of the grounding line. Here numerous sandstone cobbles are found on smooth, striated granitic bedrock. We collected quartz-rich cobbles resting on glacially eroded bedrock, along four separate altitudinal transects (Supplementary Fig. 1). The Mt Suess/Gondola Ridge (upper) transect included 16 sampled erratics at 824–587 m a.s.l. (260–24 m above the modern ice surface), while an additional 18 samples were collected as part of two further transects at the downstream end of Gondola Ridge (mid-lower and lower). At Low Ridge, 10 samples were collected with an elevation range of 264–62 m a.s.l. (204–2 m above the modern ice surface).

Cosmogenic nuclide surface-exposure dating allowed us to establish a chronology of ice surface lowering (see Methods). Across all 4 transects, 44 exposure ages ranged from 22.3±2 thousand years to 245±75 years BP (Supplementary Data 1; Fig. 2). Only four sample outliers were identified in the complete data set, by their stratigraphic positions within and between transects (Methods). The remaining 40 samples provide a direct constraint on outlet glacier thinning from the LGM to preindustrial times, with 29 of these samples revealing an episode of rapid Holocene thinning. The high coherence and sample density of our cosmogenic exposure ages with few outliers allows us to refine the timing of former ice surface lowering at Mackay Glacier using Bayesian age-elevation modelling (Methods).

The resulting chronology provides a near-complete record of outlet glacier thinning, with the Mt Suess/Gondola Ridge (upper) transect revealing ice surface lowering from ∼22 ka BP into the late Holocene (Fig. 3a–c). Undated glacial erratics occur on Mt Suess up to 340 m above the modern ice surface (∼890 m a.s.l.), indicating that Mackay Glacier may have been this thick during the LGM, or at another time in the recent geological past. Two dated samples from a surface elevation ∼260 m above the modern glacier date to the LGM (∼22.3±2 and 18.3±3.5 ka BP). Subsequent ice surface lowering below this elevation appears to have been initially gradual, with just ∼30 m of thinning occurring before ∼8 ka BP. However, this part of the record lacks sufficient sample coverage to clarify surface elevation changes that might have occurred between the LGM and early Holocene. The most notable ice surface elevation changes occurred in the mid-Holocene, when >80% of the total LGM-to-present thinning is recorded. This event is best preserved in the Mt Suess/Gondola Ridge (upper) transect, where rapid thinning is recorded from 230 to 50 m above the modern ice surface (180 m total lowering). The onset of rapid thinning occurred by ∼6.8 ka BP (see Methods; Supplementary Fig. 2). The end of this thinning event is likely recorded by five exposure ages at Gondola Ridge (lower), which is shown after ∼6 ka BP as a return to gradual rates of thinning. Data from low ridge show that this more gradual thinning during the mid-late Holocene (a further ∼25 m of ice surface lowering) persisted until ∼250 years ago, at least in the lower reaches of the glacier.

To estimate the rate and duration of rapid thinning at Mackay Glacier, we carried out linear regression analysis on data from the Mt Suess/Gondola Ridge (upper) and Low Ridge transects. While the peak rate of surface lowering during this episode and the corresponding duration are not possible to determine, this analysis provides the most probable time-averaged estimates for the whole episode of rapid thinning. Regression was applied randomly to exposure ages through a Monte Carlo simulation (see Methods; Supplementary Fig. 3). First, we assessed the rate of thinning, assuming a linear rate implied by the samples. Previous authors have used raw exposure ages and associated uncertainties for regression analysis to derive thinning rates^19^. Applied to Low Ridge, this regression analysis indicates thinning at 8.2–358.8 cm per year (2 σ). Due to the higher sample density at Mt Suess/Gondola Ridge (upper, *n*=6), we were able to use the Bayesian-modelled ages to estimate thinning rates, which we consider to provide the best estimates of the surface lowering history. Here regression analysis indicates thinning of 33.1–80.2 cm per year (2 σ) and, as the rate likely varied during this period, we suggest that the upper end of this range better represents the peak of thinning (Fig. 3d). Second, the regression analysis also provides an estimate for the duration of this rapid thinning episode (Methods; Fig. 3e). At Mt Suess/Gondola Ridge (upper), the duration of this event is constrained by a dominant, normally distributed peak at ∼420 years (251–731 years, 2 σ), which also corresponds to its median and mean values. At Low Ridge, a wide and skewed distribution of possible durations are estimated with a best fit of ∼300 years; however, the median value is consistent within 1 σ of Mt Suess/Gondola Ridge (upper) at ∼400 years. We consider these to be minimum estimates of duration as the onset of rapid thinning is not recorded at Mt Suess/Gondola Ridge (upper), while neither the start nor end is recorded at Low Ridge.

In summary, Bayesian age-elevation modelling and regression analysis of ^10^Be exposure ages demonstrate that an episode of accelerated ice surface lowering occurred at Mackay Glacier during the Holocene. This thinning was rapid both in the context of its LGM-to-present deglaciation history and of rapidly changing outlet glaciers observed in Antarctica today, such as Totten and Pine Island Glaciers whose margins are thinning at ∼40–150 and ∼50–>200 cm per year, respectively^9^. At Mackay Glacier, this episode of surface lowering persisted for at least 251 years (95% confidence), providing a window into ice sheet behaviour that extends far beyond the period of modern satellite observations.

### Absence of large climatic or oceanic changes in the Holocene

Substantial, rapid and prolonged ice surface lowering at Mackay Glacier occurred during a period when proxy data indicate an absence of large climatic or oceanic changes, especially in the context of LGM-to-present environmental changes (Fig. 3f). The Holocene climatic optimum in Antarctica was reached at ∼10–12 ka BP (ref. 20), and ice core records show that atmospheric temperatures cooled slightly from this peak to reach conditions similar to preindustrial climate by ∼7,000 years ago^21^,^22^. Rates of eustatic sea level rise reduced significantly after ∼8 ka BP (ref. 23), and the small amount of continuing sea level rise was most likely buffered at the Antarctic coastline by regional isostatic uplift^24^. This probably resulted in a lowering relative sea level in the region of Mackay Glacier during this time^25^. Although we lack proximal information about oceanic conditions, it also seems unlikely that very large changes in ocean temperature occurred at the time of rapid surface lowering, as far-field ocean temperature reconstructions show little change during this period^26^; sea surface temperatures in the southwest Pacific Ocean also peaked before 10 ka BP, during the early Holocene climatic optimum. Therefore, while small-scale fluctuations in any of these variables could have influenced the precise timing of initial retreat^8^, external forcing from the atmosphere or ocean alone cannot explain the rapid nature of recorded ice surface lowering at Mackay Glacier.

### Deglaciation in the western Ross Sea

**Figure 1:**
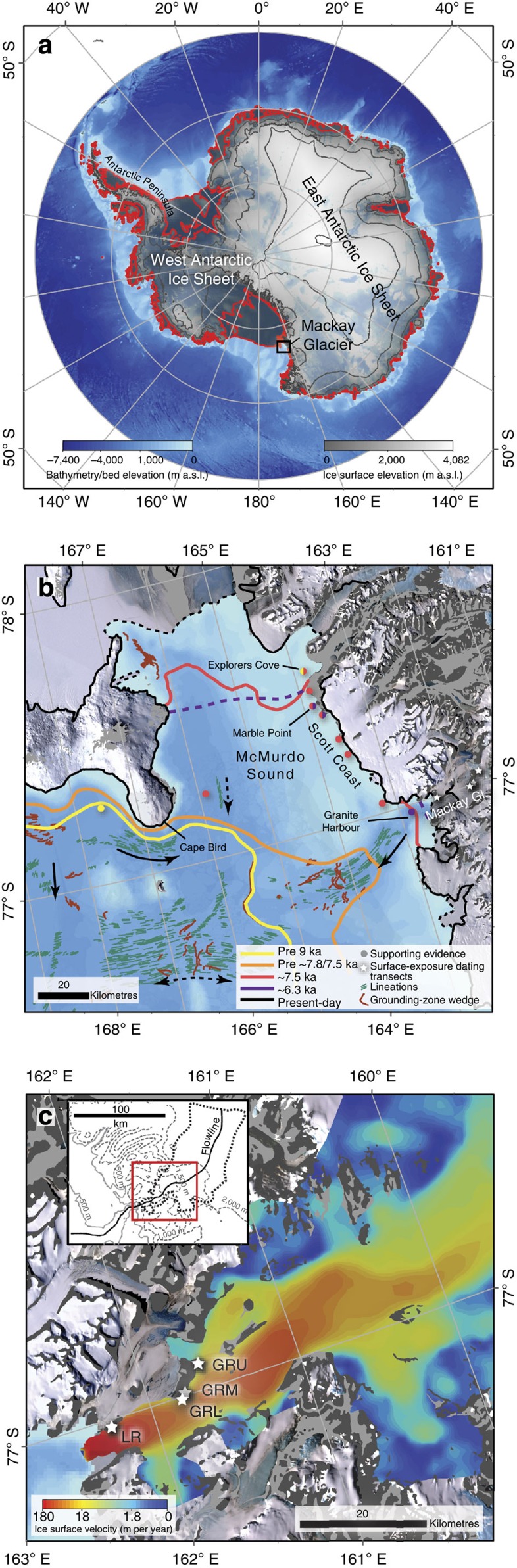
Study context. (**a**) Ice shelves (outlined in red) buttress parts of Antarctic ice sheets that are grounded below sea level. Mackay Glacier and the location of **b** is marked by a black box. (**b**) Reconstructed grounding line (solid lines) and ice shelf (dashed lines) retreat downstream of Mackay Glacier, supported by local chronological evidence and offshore geomorphology (Supplementary Table 1). Arrows denote inferred flow of past grounded ice. (**c**) Surface velocity^18^ of present-day Mackay Glacier catchment with starred sample transect locations at Mt Suess/Gondola Ridge (upper, mid-lower and lower; GRU, GRM and GRL) and Low Ridge (LR), adjacent to the main flow path. Inset shows the flowline used in model simulations and surface contours.

**Figure 2:**
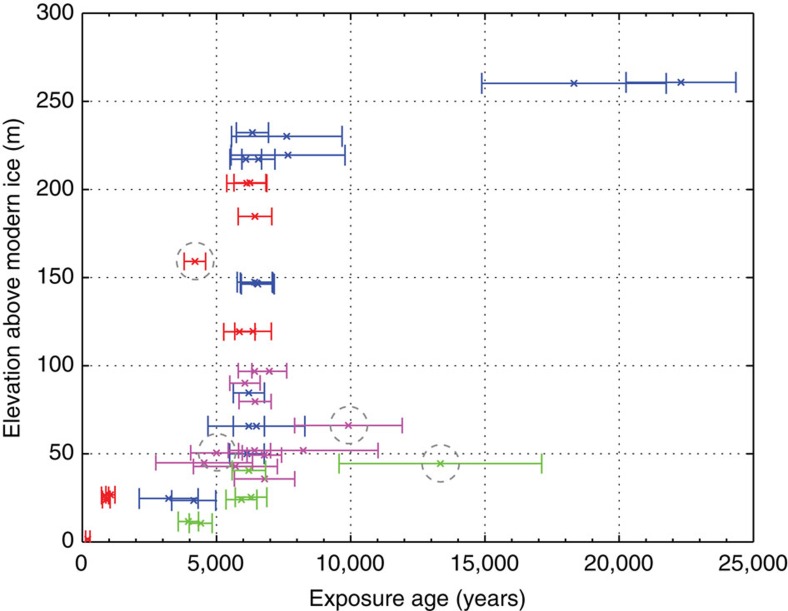
Surface-exposure ages from altitudinal transects at Mackay Glacier. Displayed are the raw, unmodelled ages (1 σ) with outliers included and identified (dashed circles). Surface lowering is recorded from ∼22 ka to ∼200 years ago with an episode of rapid thinning evident in all four transects, Mt Suess/Gondola Ridge upper (blue), Gondola Ridge mid-lower (purple) and lower (green), and Low Ridge (red).

Independent geological evidence constrains the timing and extent of past ice retreat in the western Ross Sea (Fig. 1b; Supplementary Table 1; Supplementary Note 1). Large-scale grounding line and ice shelf retreat to just north of Ross Island occurred by ∼10 ka BP (ref. 27), or perhaps slightly earlier (Supplementary Note 1). At ∼9.4 ka BP, Explorer's Cove was still occupied by grounded ice^28^ and Hall *et al*.^25^ suggest grounded ice in McMurdo Sound had yet to retreat. A relative sea level curve produced from radiocarbon dating of raised beach deposits indicates that final unloading of grounded ice adjacent to the Scott Coast occurred at ∼7.5 ka BP (ref. 25), which is supported by a radiocarbon dated bivalve in a sediment core collected off Cape Bird^29^. The remaining ice shelf disappeared between ∼7.5 and ∼6.3 ka BP, at which point Granite Harbour^30^, Gneiss Point and Marble Point^28^,^31^ had become open water. An ice shelf may have, however, still existed immediately south at Explorer's Cove^28^.

The geomorphology on the present-day seabed helps infer the style and rate of past grounded ice retreat in and around McMurdo Sound during the Holocene^15^. Immediately, downstream of Mackay Glacier's terminus is the deep Mackay Sea Valley (below −800 m a.s.l.), which shallows and widens into a trough and then the southern Drygalski Basin, north of McMurdo Sound (Supplementary Fig. 4). A series of grounding-zone wedges (GZWs) preserved in the outer part of the trough imply that the initial retreat of the Mackay Glacier grounding line was staggered.

Our surface-exposure chronology from Mackay Glacier indicates that rapid, uninterrupted thinning occurred over at least two and a half centuries. This most likely happened after the glacier retreated from the GZWs of the outer trough to the inner parts of Mackay Sea Valley, during a time when the large buttressing effect provided to Mackay Glacier by grounded ice, and possibly an ice shelf, in the Ross Sea was removed. Offshore chronologies indicate that these conditions were met by ∼7.5 and ∼6.3 ka BP, respectively, which is consistent with the timing of rapid thinning recorded in our onshore chronology (Supplementary Fig. 2).

Dynamic glacier thinning requires a reduction of resistive stresses at the bed, grounding line, or near the terminus^7^,^12^,^32^,^33^. Regional geological evidence indicates that the most probable processes that drove rapid thinning of Mackay Glacier were retreat over a reverse bed slope into Mackay Sea Valley and/or removal of a local ice shelf, providing a testable hypothesis.

Bathymetric profiles of Mackay Sea Valley and trough reveal an overdeepening, which could have resulted in Mackay Glacier retreating as a consequence of marine ice sheet instability^2^. The loss of buttressing from ungrounding at the terminus during periods of rapid grounding line retreat produces a dynamic adjustment where thinning is propagated upstream^7^,^33^,^34^. The confined lower reaches of Mackay Glacier during the mid-Holocene were most likely susceptible to such upstream propagation; today, surface velocities of >150 m per year^18^ and probable abundant basal sliding^35^ help to quickly redistribute ice mass^7^. Abundant roche moutonées and striae on Cuff Cape near the current terminus indicate that these warm-based conditions also existed in the recent past^36^.

Geomorphological evidence from the region suggests that Mackay Glacier may have also been influenced by the loss of an adjacent ice shelf. Ice shelf presence may have acted to slow or halt retreat at Mackay Glacier during its earliest stages, even if the grounding line was positioned on a reverse bed slope^5^,^11^,^37^. Some GZWs are located on a reverse bed slope within the Mackay Glacier trough (Supplementary Fig. 4), possibly suggesting that a supporting ice shelf was present at this time. The ultimate loss of this ice shelf would have led to increased ice fluxes over the Mackay Glacier grounding line^38^, possibly enabling a period of accelerated retreat and the rapid thinning recorded onshore.

Both of these scenarios involve rapid thinning of Mackay Glacier being driven by perturbations near its terminus, resulting in glacier retreat through an overdeepened trough.

### Numerical modelling of glacier retreat and surface lowering

In an attempt to understand whether progressive retreat of Mackay Glacier through its overdeepened trough resulted in the rapid thinning recorded, we simulated the glacier using a one-dimensional flowline model (see Methods). First, we simulated the modern ice surface at the sampling transects with a grounding line located upstream of the large overdeepening, and an advanced glacier that extended beyond the most distal GZW immediately offshore from Mackay Glacier and which honoured our empirically derived estimates of ice thickening along its longitudinal profile.

To assess whether the change in surface elevation at our thinning transects could be explained simply by recession of the grounding line, we forced the grounding line from the outer trough, through the zone of GZWs and across the reverse bed slope of the inner trough. This was achieved using a range of individual ocean temperature and calving rate scenarios as the forcing. We also investigated the effects of pulses of sea level rise on the glacier, for example, if the rate of eustatic sea level rise exceeded that of isostatic rebound. In all experiments, we were able to simulate retreat from the outermost GZW to upstream of the overdeepened Mackay Sea Valley in ∼300–400 years, in good agreement with our chronology (Fig. 4; Supplementary Fig. 5a).

**Figure 3:**
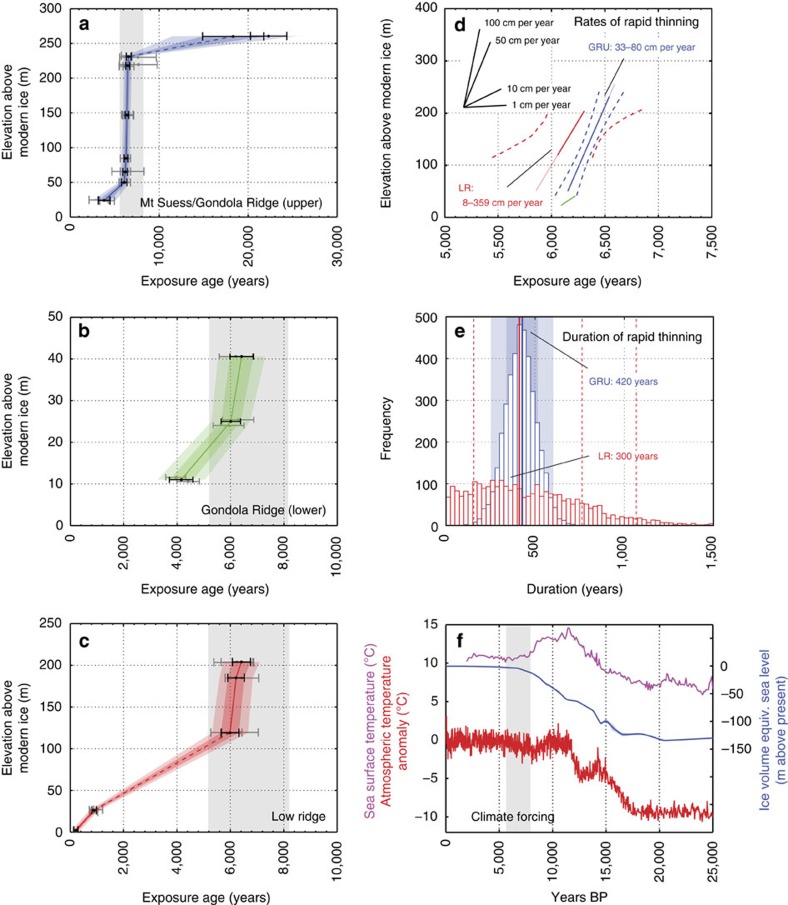
Age-modelled chronology of surface lowering at Mackay Glacier and regional climate forcing. Bayesian age-elevation modelled surface-exposure ages (black, 1 σ) are shown for three of four transects (**a**–**c**), linearly interpolated with 1 and 2 σ uncertainty. Initial raw exposure ages (Fig. 2) are in grey. Rapid thinning is recorded at ∼6,800-6,000 years before present. (**d**) Rapid surface lowering is estimated at Mt Suess/Gondola Ridge (upper; GRU) and Low Ridge (LR), assuming linear thinning rates between ∼6.8 and 6.0 ka (Supplementary Fig. 3). GRU provides a tighter estimate of 33.1-80.2 cm per year. The best fit (solid lines) and 95% confidence bounds (dashed lines) are shown. At Low Ridge, the full extent of rapid thinning is uncertain; however, the end of this episode and a change to more gradual thinning may be recorded at Gondola Ridge (lower; green line). (**e**) Rapid thinning lasted for ∼420 years (251-731 years, 2 σ), based on the higher-quality GRU chronology. As the onset of rapid thinning here may have been from the LGM surface elevation, we consider this a minimum estimate for the full duration of the episode. Median estimates are shown by solid lines, while 1 and 2 σ are denoted by a shaded area (GRU) and dashed lines (LR). (**f**) This episode does not correspond to significant increases in regional sea surface temperature^26^, atmospheric temperature^21^ or sea level^23^, irrespective of exposure age uncertainty (grey shaded area; Supplementary Fig. 2).

**Figure 4:**
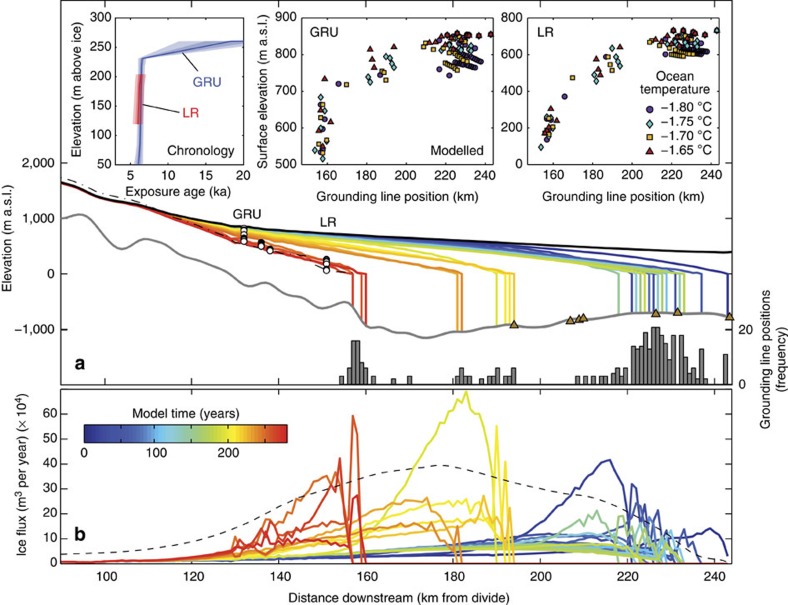
Modelled retreat of Mackay Glacier through the overdeepened trough with corresponding accelerated ice flux and upstream thinning at our transects. (**a**) An evolving surface profile is shown at 10-year intervals for one model run (−1.75 °C ocean temperature forcing scenario). The modern surface (dot-dashed line), simulated advanced profile (bold black line) and sample locations (white circles) are also shown. A histogram displays the frequency of grounding line positions for all modelled scenarios, with peaks broadly corresponding to most grounding-zone wedges (brown triangles). This modelling identifies that stable glacier positions occur in the outer trough, unstable fast retreat occurs on the reverse bed slope and then restored stability occurs in the vicinity of the present-day grounding line. (**b**) An evolving ice flux corresponds to the surface profile. As the grounding line retreats over the reverse bed slope, thicker ice allows for greater ice fluxes and therefore potential for mass loss. The largest flux of ice occurs in the deepest part of the trough, where the glacier is thickest and a steep ice surface slope facilitates higher velocities. Ice flux remains relatively large until the glacier stabilises on the normal-sloping bed, temporarily maintained by high surface velocities. The dashed black line denotes the longitudinally averaged (25 km) ice flux (5 × 10^4^) over this retreat period. Simulated surface thinning at our Mt Suess/Gondola Ridge (upper; GRU) and Low Ridge (LR) transects is shown in scatter plots (**a**) for a range of ocean forcing scenarios. Some initial thinning occurs in all scenarios and at both transects when the grounding line is located in the outer trough. Accelerated surface lowering occurs first at LR simultaneous with retreat over the reverse bed slope, while the most rapid thinning is simulated upstream at GRU, matching our surface lowering chronologies.

In all retreat scenarios, the grounding line fluctuates in the outer trough, retreats more rapidly over the reverse bed slope and then re-establishes stability on the normal slope of the bedrock ridge upstream of the overdeepening (Fig. 4a). A higher frequency of grounding line positions were simulated in the outer trough, which in most cases match observed GZWs^15^. In the overdeepening, retreat into deeper water allows for thicker ice at the grounding line that is closer to floatation, leading to greater ice fluxes and therefore further retreat (Fig. 4b). While grounding line retreat is generally faster for warmer ocean temperatures and higher relative sea level, the pattern of upstream thinning is similar in all forcing scenarios (Fig. 4a; Supplementary Fig. 5a); accelerated upstream thinning occurs as the grounding line retreats over the reverse bed slope. The increased rate of surface lowering is initially simulated at Low Ridge. Subsequently, a more pronounced thinning response occurs at Mt Suess/Gondola Ridge (upper). This spatial pattern of ice-dynamic thinning fits the thinning gradients of our respective transect chronologies (Fig. 3) and the idea that thinning propagated upstream following perturbations at the grounding line^11^,^33^,^39^. In particular, prolonged thinning that still occurs when the grounding line is located on the normal bed slope may reflect the steep surface slope in this portion, which is able to facilitate high ice velocities and maintain ice loss^40^.

A rapid episode of dynamic thinning in our model may have occurred due to a process other than that provided by the reverse bed slope. Therefore, we also carried out an idealized experiment in which Mackay Sea Valley was replaced by a flat bed (Supplementary Fig. 5b). Under this modified bed geometry, modelled retreat occurs over a longer period of ∼800 years without substantial jumps in the grounding line position. The e-folding response time^41^ of the glacier for this experiment is 589±45 model years, opposed to the faster response of 290±126 model years when ice retreated through the overdeepened trough. Importantly, there is no increase in the rate of surface lowering in this simulation, and it therefore fails to replicate the rapid thinning recorded in our Mt Suess/Gondola Ridge (upper) and Low Ridge transects.

In summary, simulated accelerated thinning corresponds with increased ice flux as the grounding line retreats over the reverse bed slope, irrespective of the forcing applied. Together, the modelling experiments and regional geological constraints suggest that the substantial thinning recorded at our nunatak sites most likely resulted from marine ice sheet instability^2^,^4^,^5^. Reduced buttressing from ice shelf disintegration^5^,^42^ is not required to explain rapid retreat and dynamic thinning but it could have helped facilitate this retreat.

## Discussion

Rapid and protracted ice loss of marine-terminating glaciers that retreat into deeper water has been observed in temperate and sub-polar settings^7^,^13^,^43^,^44^,^45^. Similar to Mackay Glacier, rapid retreat and ice-dynamic thinning becomes self-sustaining in these glaciers as the grounding line recedes across a reverse bed slope^8^,^33^,^46^. In these environments, a combination of atmospheric and oceanic warming, and rising sea level may produce a succession of negative mass balance years that is sufficient to initiate topographically driven retreat^8^,^47^. Today, accelerated ice loss occurs where warm subsurface waters infiltrate glacial troughs, such as at Jakobshavn Isbræ and Pine Island Bay^48^,^49^. The record at Mackay Glacier demonstrates that unstable tidewater glacier retreat via the marine instability mechanism is also possible in a more polar oceanic environment, >600 km from the Antarctic continental shelf edge. Our modelling indicates that small changes in ocean temperature and/or sea level rise were sufficient to initiate prolonged retreat into deeper water with corresponding dynamic thinning and ice loss.

Most tidewater glacier cycles have been observed to last for up to several decades as a result of small variations in the bed topography^7^,^13^,^44^,^45^. Our chronology provides a time-averaged estimate of ice-discharge events and ice-dynamic thinning for the retreat phase of a prolonged tidewater glacier cycle. The scale of this outlet glacier retreat is similar to that observed in the twentieth and early twenty-first centuries in well-known temperate or sub-polar tidewater environments, such as Glacier Bay, Alaska and Jakobshavn Isbræ, Greenland, which have retreated ∼100 and 35 km, respectively, between ∼1850 and 2010 AD^50^,^51^,^52^,^53^. However, at Mackay Glacier, we show that this retreat and associated accelerated ice surface lowering continued for at least two and a half centuries, providing a record of tidewater glacier behaviour that extends over a longer period than historic observations. The timescale of this retreat is independently supported by the modelled response time of the glacier (∼300 years).

Glacial troughs similar to Mackay Sea Valley (excavated to ∼1 km below sea level or greater) and their associated reverse bed slopes are common features on the inner continental shelf surrounding Antarctica^1^,^54^. Ice-dynamic thinning and rapid mass loss may therefore have occurred in many parts of Antarctica, as grounding lines retreated through these troughs. Marine grounding lines retreated from the outer to inner continental shelf following the LGM, where they encountered troughs with reverse bed slopes^54^,^55^. We suggest that phases of accelerated ice sheet thinning during the Holocene in West Antarctica and on the Antarctic Peninsula^17^,^19^,^56^,^57^,^58^ may also have resulted from the topographic instability that drove the retreat of Mackay Glacier.

Our new data from East Antarctica reveals that accelerated ice sheet thinning as a consequence of marine ice sheet instability can lead to hundreds of metres of ice surface lowering. We highlight that periods of rapid, topographically driven ice loss are likely typical of marine ice sheet margins, even in East Antarctica. These findings provide confidence for models that are able to simulate such ice-dynamic retreat^14^,^59^,^60^ and which predict centennial-scale responses in locations where overdeepened basins extend inland for many tens of kilometres from glacier or ice sheet margins^14^.

## Methods

### Sample collection

Forty-four samples were collected for surface-exposure dating from two nunataks in the lower reaches of Mackay Glacier. The upstream nunatak comprises Gondola Ridge, an elongate granitic ridge, and Mt Suess, a basaltic dome that peaks at 1,127 m a.s.l. Low Ridge is an area of exposed granitic bedrock (∼205 m above the ice surface) that occurs downstream of the present-day grounding line. Geomorphological evidence shows that Mackay Glacier was larger in the geologically recent past^61^. Moraines at Cuff Cape, downstream of the present-day grounding line, record small fluctuations of Mackay Glacier and adjacent New Glacier termini during the last two centuries^36^. Before this, its glacial history is unconstrained, although regional-scale ice sheet reconstructions suggest that Mackay Glacier along with other outlets on the Victoria Land coast thickened at the LGM, due to buttressing by grounded ice in the Ross Sea^62^.

We carried out altitudinal sampling transects at Mt Suess/Gondola Ridge to constrain thickness at the LGM and subsequent initial thinning, and at Low Ridge to record more recent thinning near the present-day terminus (Supplementary Fig. 1). To minimize the risk of sampling clasts containing an inherited cosmogenic inventory, erratics were sampled that showed signs of glacial transport and erosion, evidenced by facetted and abraded surfaces. Erratics that rested in locations unaffected by post-depositional processes were also prioritized to provide a simple exposure signal. Such samples were generally either perched on eroded bedrock or propped up by a thin layer (1–3 cm) of draped glacial till. Samples consisted of cobbles (∼10–25 cm in length) and some small boulders (<1 m across) that were subsampled in the field. Details of samples collected are listed in Supplementary Data 1.

### Sample processing and measurement

Preparation of samples for surface-exposure dating was conducted at the sedimentology, cosmogenic nuclide and geochemistry laboratories of Victoria University of Wellington and GNS Science, New Zealand. The upper ∼3–7 cm of each sample was extracted for processing using a large circular saw. This material was then crushed with a fine jaw-crusher and sieved to retain sand-sized grains (250–500 μm). Each sample was initially cleaned using a Frantz Isodynamic separator (at ∼0.5 A and a 10° tilt) to remove the magnetic component. Further cleaning was required to remove non-quartz minerals and etch any meteoric beryllium from the outer surface of the grains. This was achieved for each sample (up to 150 g) with a 1-day leach in hydrochloric acid and then three 2-day leaches in a 5% mixture of weak hydrofluoric acid and nitric acid, warmed and rotated on hotdog rollers. A final 1-h hydrofluoric acid (7 M) leach and concentrated aqua regia cleaning was carried out to remove any remaining meteoric beryllium before sample dissolution and (*in situ*) beryllium extraction.

Beryllium was extracted following established geochemical procedures^63^. A ^9^Be spike (∼0.15–0.18 mg per sample) was added to the quartz samples, which were then dissolved in concentrated hydrofluoric acid. Anion exchange columns were first used to remove iron, and then cation exchange columns were used to isolate beryllium from iron, aluminium, titanium, sodium and magnesium. Procedural blanks yielded mean ^10^Be/^9^Be ratios of 1.38 × 10^−15^ with a s.d. of 5.91 × 10^−16^. Analytical measurements of ^10^Be/^9^Be were undertaken at ETH Zurich mass spectrometry facilities using both Tandem and Tandy accelerator mass spectrometers^64^. Low measureable yields of a small number samples resulted in less precise exposure ages with ∼17–33% uncertainties (samples GR34, GR37, GR47, GR51, GR54, GR62b, GR64 and GR83). All samples were measured relative to the ETH Zurich in house standard S2007N (nominal ^10^Be/^9^Be ratio of 28.1±0.8 × 10^−12^), which in turn was calibrated relative to the ICN 01-5-1 standard (^10^Be/^9^Be ratio of 27.09±0.3 × 10^−12^) (ref. 65), and were corrected with procedural blanks.

### Surface-exposure age calculation and outliers

Surface-exposure ages were calculated from the measured concentrations of ^10^Be, corrected for topographic shielding, sample thickness, quartz density (2.7 g cm^3^) and an Antarctic atmospheric pressure gradient, using CRONUS-Earth online calculator^66^ (Supplementary Data 1). The production rate of ^10^Be is currently unconstrained in Antarctica, and production rates from both a global data set^66^ (production due to spallation of 4.49±0.39 to 4.96±0.43 atoms g per year) and New Zealand (NZ) calibration site^67^ (3.84±0.08 atoms per gram per year) have been applied to Antarctic chronologies in the past^17^,^19^. We prioritize the global production rate in this study as the high-precision NZ production rate (44° S) would produce ages with artificially low uncertainties at Mackay Glacier (77° S), given the uncertain temporal and spatial scaling. The choice of production rate does not significantly affect the reconstructed thinning rate at Mackay Glacier, and instead primarily influences its absolute timing (Supplementary Fig. 2).

Four sample outliers were identified in the data set. On Gondola Ridge (mid-lower), two samples were erroneously old or young (GR38 and GR40), representing micro-inheritance and some post-depositional effects, respectively. At Gondola Ridge (lower), the oldest sample (13,080±3,700 years BP, GR83) is not consistent with the thinning trends recorded in adjacent transects; if the surface elevation here was <50 m above present before ∼13 ka, then an unfeasible ice surface profile would exist with sites upstream and downstream, which record glacier surfaces >200 m higher at this time. For this reason, sample GR83 most likely contains a small amount of inheritance. A single sample on Low Ridge (CC93) is not consistent with exposure ages above and below its elevation. This minor outlier has probably been affected by post-depositional rotation or spalling.

### Bayesian age-elevation modelling

We applied Bayesian modelling^68^ to refine the timing of surface lowering recorded in our high-density chronologies, adapted here for altitudinal surface-exposure transects. This approach narrows the possible age uncertainty ranges based on the respective likely age distribution and elevation, with the assumption that older and higher samples would be exposed by glacier thinning before the lower samples. At elevations where internal uncertainties overlapped, we calculated a weighted mean and standard error. We used external exposure age uncertainties that represent production rate uncertainty from spallation and muons, as we compare the output age ranges to other chronologies^66^. Although the Gondola Ridge (mid-lower) chronology is consistent with other transects, it was not possible to apply Bayesian age-elevation modelling at this site, as the ages show slightly larger stratigraphic scatter^68^. However, at Mt Suess/Gondola Ridge (upper), Gondola Ridge (lower) and Low Ridge, this statistical analysis of high-density sample transects allowed the uncertainty of raw exposure ages to be reduced (Fig. 3a–c; Supplementary Data 1).

### Regression analysis of rapid thinning episode

To estimate the rate and duration of rapid thinning at Mackay Glacier, linear regression analysis was carried out on data from the Mt Suess/Gondola Ridge (upper) and Low Ridge transects for the period of ∼6.8–6.0 ka. Error-weighted least-squares regression was applied randomly to normally distributed exposure ages (2 σ) through a 4,000-iteration Monte Carlo simulation. Rates and durations of rapid thinning were estimated from the distribution of feasible, positive-sloping linear regressions, with uncertainty generally reflective of the number of samples contributing to each transect and their respective uncertainties. Linear rates of thinning were calculated using the raw exposure ages at Low Ridge and the Bayesian-modelled ages at Mt Suess/Gondola Ridge (upper). The duration of this thinning episode was additionally determined, based on the start and end points of the modelled regressions.

### Glacier flowline model

We employed a one-dimensional finite difference flowline model to investigate whether retreat of Mackay Glacier as a consequence of grounding line retreat through an overdeepening could result in the magnitude of thinning observed at our exposure dating transects. The flowline model has previously been applied to Transantarctic Mountain outlet glaciers and is fully described in Golledge and Levy^69^. Ice thickness change is determined using the mass conservation equation, as the balance between ice flux and net accumulation, which is then integrated through time. Zero ice flux is prescribed at the top of the domain. The shallow-ice approximation is used in combination with a longitudinal averaging scheme to calculate basal shear stress at 1-km horizontal resolution. As the past ice shelf extent is not well constrained in time or space, we only implement the numerical scheme upstream of the grounding line and do not simulate an ice shelf. Basal sliding occurs where basal temperatures are close to melting point, while flow through creep is controlled by a temperature-dependent deformation rate with an enhancement coefficient. Here we used a spatially variable basal sliding coefficient to account for differences in basal traction between the higher elevation Transantarctic Mountains portion, where Mackay Glacier is currently grounded on bedrock, and the downstream marine portion, where soft sediments occur. Enhanced sliding in the marine portion is suggested by the occurrence of elongate glacial lineations in the Mackay Glacier trough (Supplementary Fig. 4), indicative of former fast ice flow from subglacial deformation over softer sediment.

Net mass balance is determined using a positive degree-day scheme, with mean surface air temperature, annual temperature range and precipitation rate initially prescribed. Surface melt is calculated if mean surface temperatures are above the freezing point. Although ice shelves are not directly incorporated into the model, negative mass balance is imposed in areas where the glacier bed is below sea level. Sub-shelf melting is calculated using an ocean temperature-based melt scheme when the ice thickness at the grounding line is less than floatation thickness^69^, controlled by a given seasonal ocean temperature cycle. Otherwise, mass loss at the grounding line occurs from tidewater calving using a scalable calving rate coefficient as a factor of a calving constant^69^.

The model was initialized with bed topography and ice thickness obtained from multiple radar data profiles. Glacier surface elevations were obtained from airborne altimetry measurements Investigating the Cryospheric Evolution of the Central Antarctic Plate (ICECAP), while the bed topography was interpolated from BEDMAP2 data^1^ that was corrected along the flowline with radio-echoed ice thickness measurements^70^. The model domain extends over 244 km from the ice divide near Taylor Dome to the most distal offshore GZW (Fig. 1c; Supplementary Fig. 4). The flowline used for Mackay Glacier followed the sinuous yet laterally constrained flow path through the Transantarctic Mountains, and was determined from present-day surface velocities, surface flow stripes, offshore lineations and trough bathymetry. To evaluate the thinning response resulting from migration of the grounding line without effects from lateral variations^39^, we applied a uniform width of 8 km along the length of the model domain, which approximates the glacier width in the lower reaches of the modern Mackay Glacier and the offshore trough.

Model experiments used climate and glaciological parameterisations from field measurements and commonly used physical values (Supplementary Table 2). First, modern and advanced steady-state ice surface profiles were simulated by adjusting the precipitation at sea level, precipitation lapse rate, calving coefficient, ocean temperature, and basal sliding and creep enhancement factors. A more extensive ‘advanced' glacier was simulated with the grounding line beyond the end of the domain and the surface elevation <100 m above the Mt Suess/Gondola Ridge transect, where an LGM ice surface is recorded prior to rapid thinning. This advanced state was simulated by decreasing both the calving coefficient and ocean temperature while maintaining the other tuned parameters of the present-day simulation.

Transient simulations were then carried out by forcing retreat of the grounding line from the outer trough. Glacier retreat was achieved for a range of scenarios by enhancing the calving coefficient and summer ocean temperature. No retreat scenarios simulated ice thicker than floatation with a grounding line positioned within the trough in the model domain, and therefore all negative changes in mass balance were achieved through basal melt at the grounding line. Further model experiments used increased sea level to force retreat of the glacier; however, a similar pattern of retreat and thinning was observed irrespective of environmental forcing. In all scenarios, the grounding line initially fluctuates in the outer trough, but then accelerates over the reverse bed slope, corresponding to increased ice flux and rapid glacier thinning. In some cases (for example, at ∼210 km from the divide), temporary grounding line stability that is implied by the presence of GZWs is not simulated in any retreat scenarios. This could be because of reduced trough width and complex lateral drag in this location (Supplementary Fig. 4), a stability feedback provided by the positive relief of GZW deposition, and/or enhanced stability from the presence of an ice shelf at that time, none of which were incorporated within the model.

## Additional information

**How to cite this article:** Jones, R. S. *et al*. Rapid Holocene thinning of an East Antarctic outlet glacier driven by marine ice sheet instability. *Nat. Commun.* 6:8910 doi: 10.1038/ncomms9910 (2015).

## Supplementary Material

Supplementary Information Supplementary Figures 1-5, Supplementary Tables 1-2, Supplementary Note 1 and Supplementary References

Supplementary Data 1 Surface-exposure dating sample details and ages from Mackay Glacier. Ages from each transect have been calculated using a global production rate9 with a range of production rate scaling schemes, as well as using a New Zealand (NZ) calibration site production rate10 with the time-dependent (t-d) scheme. Best estimates are derived from Bayesian age-elevation modelling, which includes weighted mean ages for samples at equal elevations (see Methods). Samples identified as outliers are shown in grey italics.
