# Supplementary material for: Rapid Holocene thinning of an East Antarctic outlet glacier driven by marine ice sheet instability
Source: Nat Commun. 2015 Nov 26;6:8910. doi: 10.1038/ncomms9910 (PMC4674764; doi:10.1038/ncomms9910)
Supplement: Supplementary Information — Supplementary Figures 1-5, Supplementary Tables 1-2, Supplementary Note 1 and Supplementary References [file ncomms9910-s1.pdf]

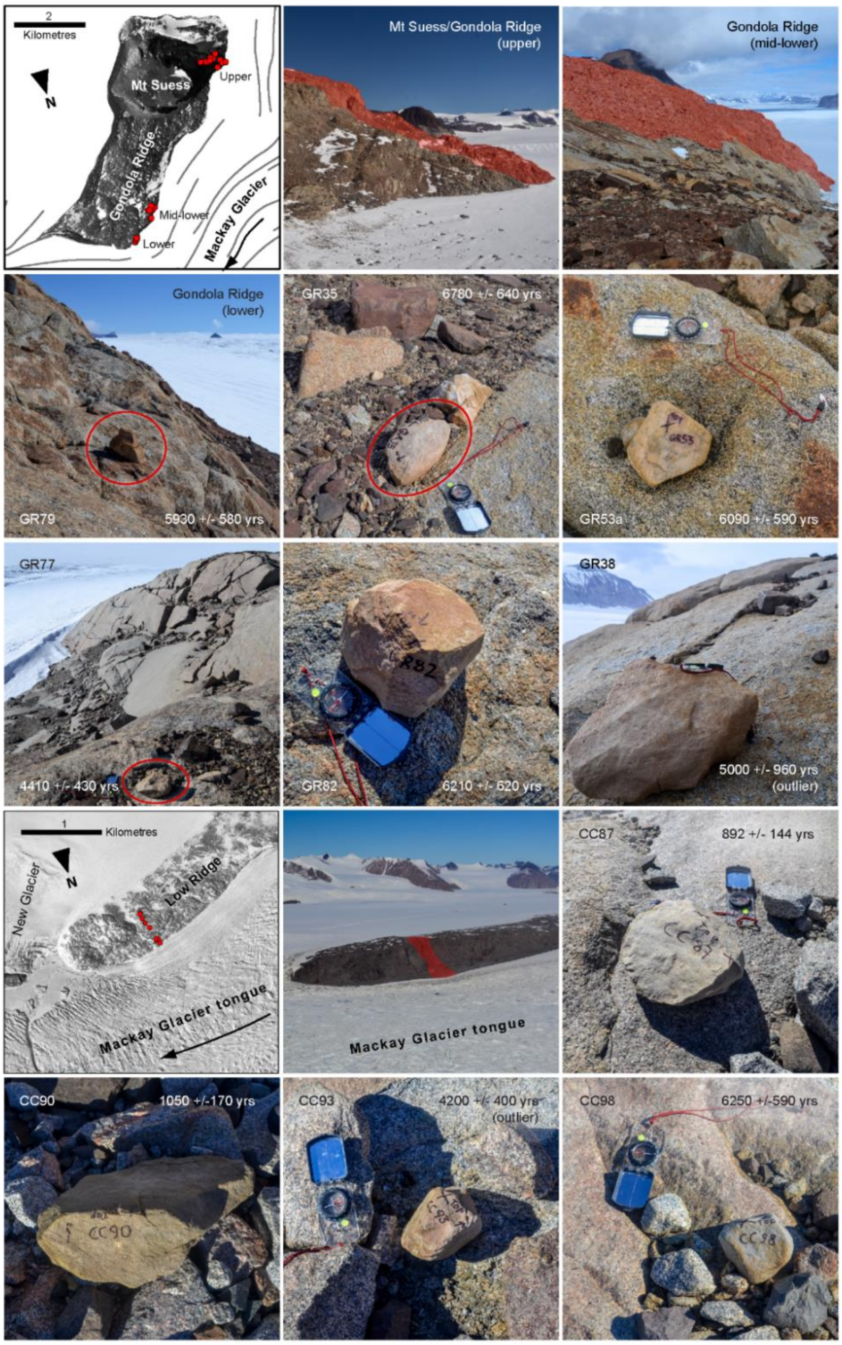

Supplementary Fig. 1. Locations of thinning transects and photos of example samples. Mt Suess/Gondola Ridge transects extended 260–24 metres above the modern surface of Mackay Glacier, and included 16 (upper), 12 (mid-lower) and 6 (lower) sampled erratics. At Low Ridge, 10 samples were collected with an elevation range of 204–2 m above the ice surface.

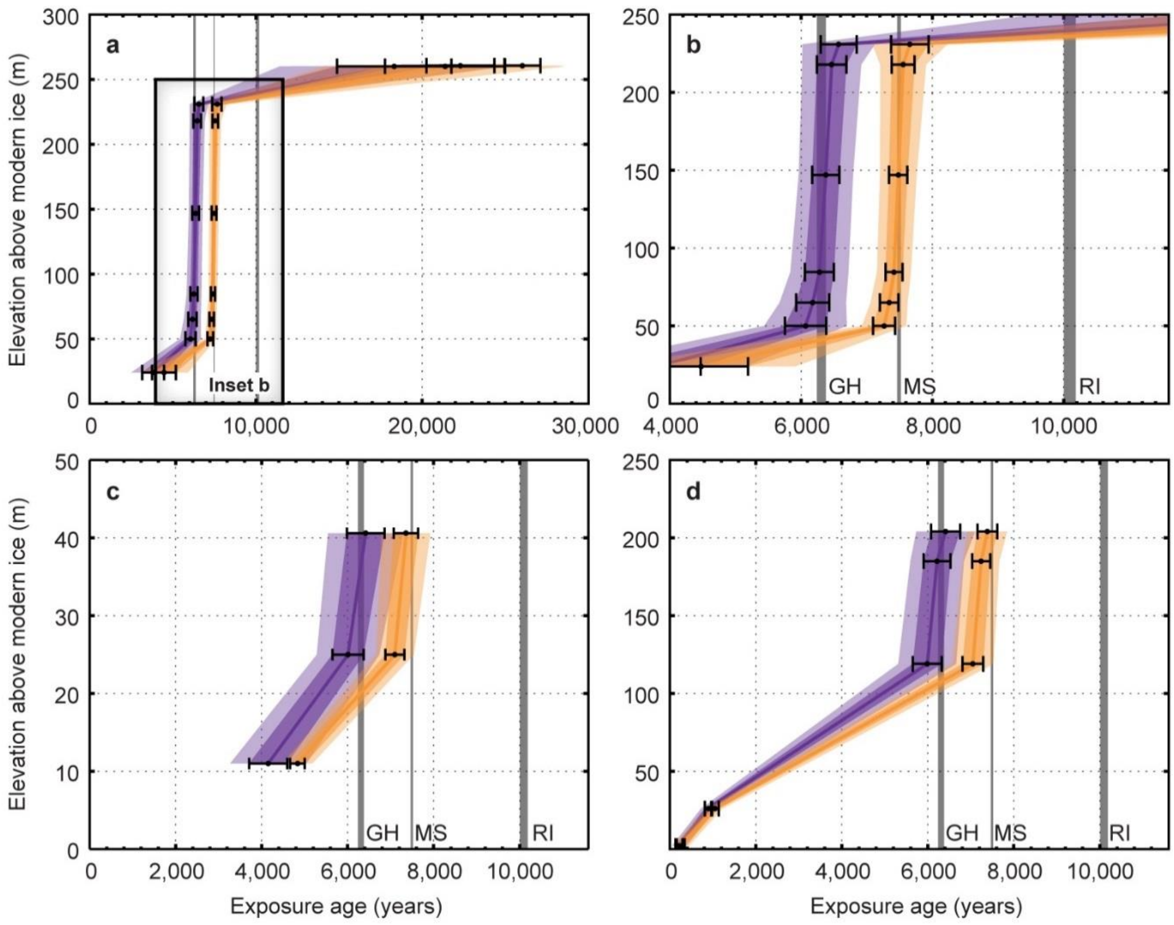

Supplementary Fig. 2. Thinning of Mackay Glacier shown for the 3 most coherent transects ((**a**) Mt Suess/Gondola Ridge upper, with (**b**) as an inset of (**a**); (**c**) Gondola Ridge lower; (**d**) Low Ridge). Displayed are Bayesian age-elevation modelled ages (1 σ), linearly interpolated between sample elevations (1 and 2 σ). The ages were initially calculated with both the global production rate[9] (purple) and New Zealand calibration site production rate[10] (orange), which can be considered as probable minimum and maximum estimations of the absolute ages. Vertical grey bars denote minimum age markers of grounded ice retreat north of Ross Island (RI), in McMurdo Sound (MS) and of ice shelf removal in Granite Harbour (GH).

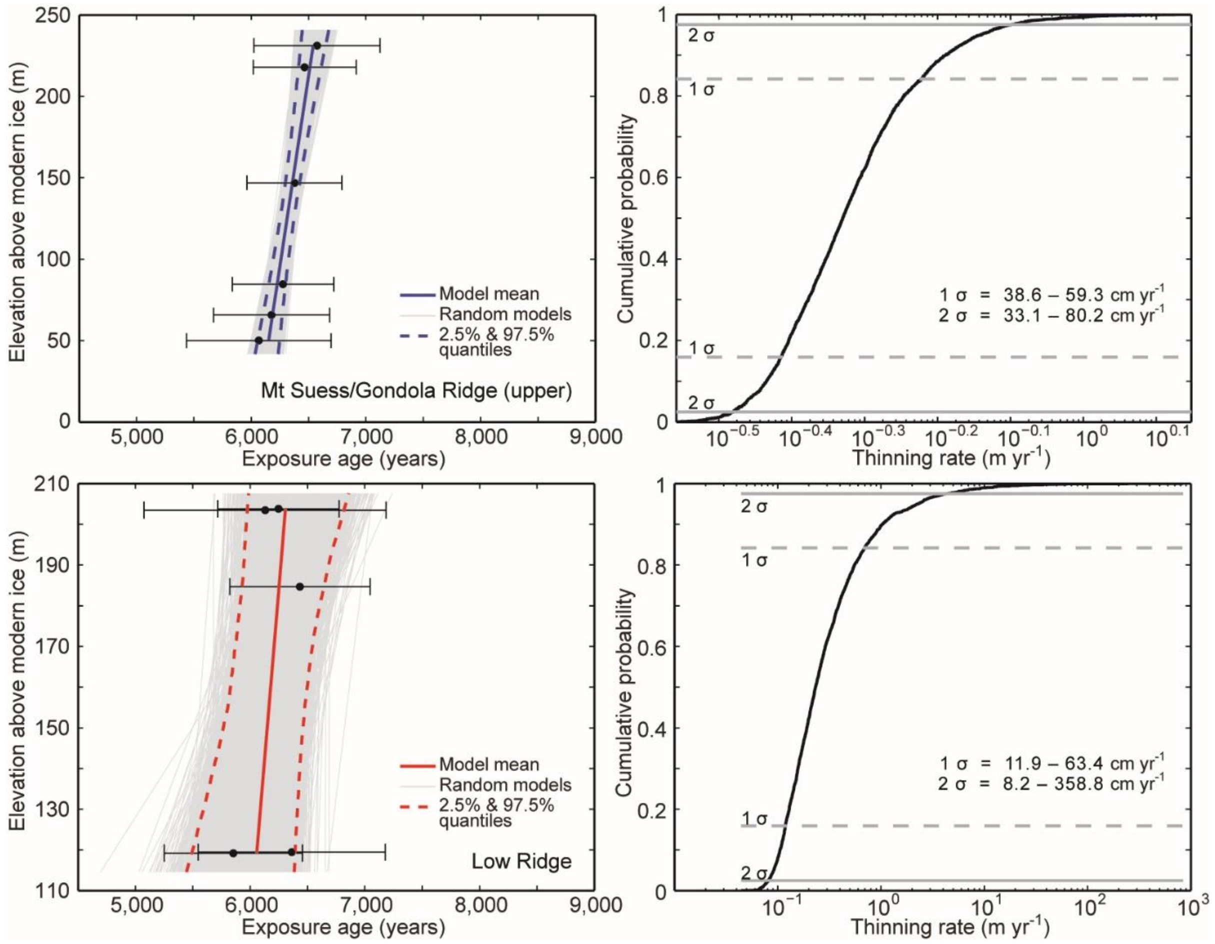

Supplementary Fig. 3. Estimated thinning rates of Mackay Glacier at Mt Suess/Gondola Ridge (upper) and Low Ridge. **Left** panels show exposure ages (2 σ) and modelled thinning profiles generated from Monte Carlo regression analysis for each transect respectively. **Right** panels show the corresponding thinning rate probability distributions. Thinning at Mt Suess/Gondola Ridge (upper) was estimated using Bayesian age-modelled ages and uncertainties, while at Low Ridge raw ages were alternatively used in regression analysis.

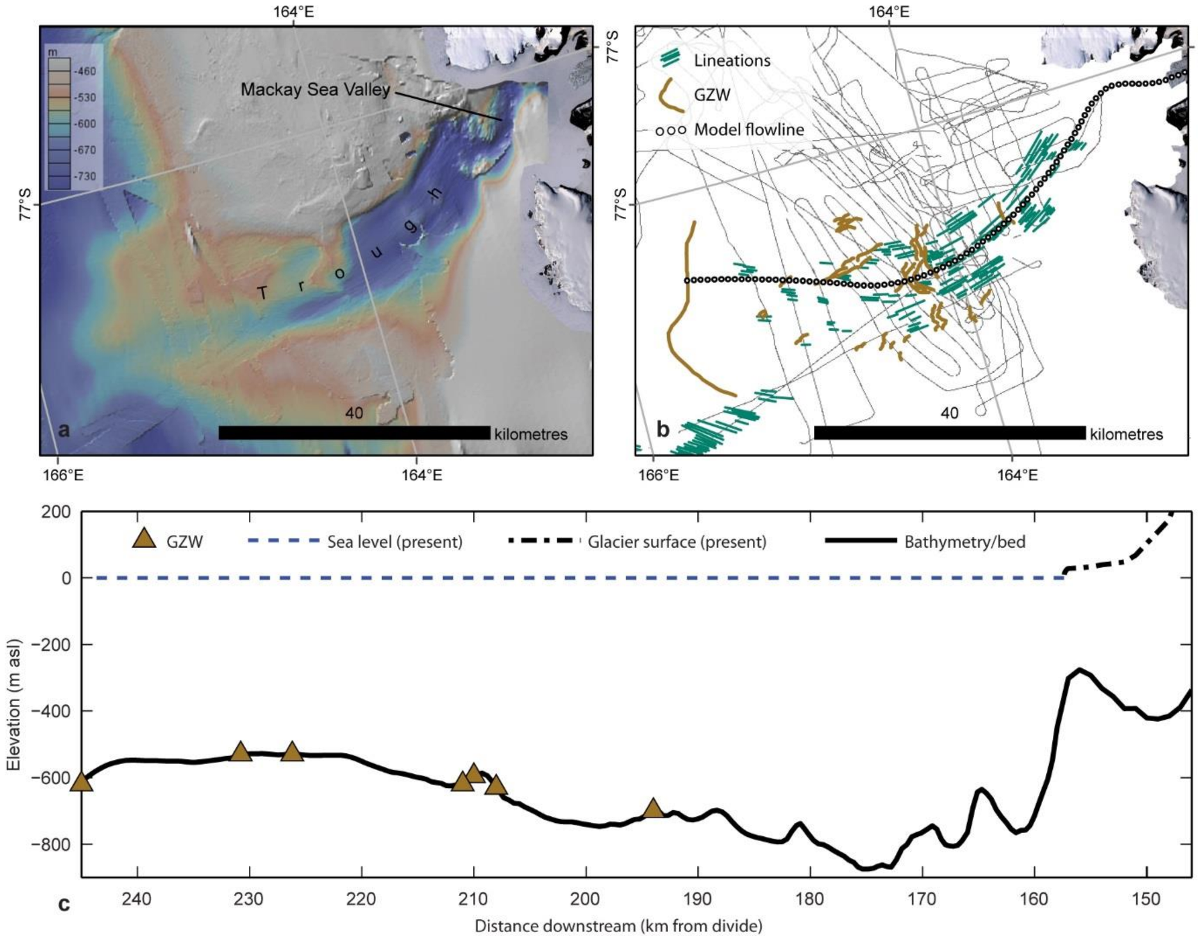

Supplementary Fig. 4. Staggered grounding line retreat and reverse bed slope of Mackay Glacier offshore trough. GeoMapApp multi-resolution bathymetry (**a**) reveals lineations and grounding-zone wedges (GZW) mapped in (**b**)[11]. High resolution multibeam tracks and the flowline (1 km horizontal resolution) used in model experiments are also shown. (**c**) Longitudinal profile of Mackay Glacier trough from high resolution bathymetry data, with GZWs indicating staggered retreat upstream of the overdeepening. Bed elevation beneath the tongue was obtained from geophysical surveys[12], and the ice surface comes from airborne radar (ICECAP).

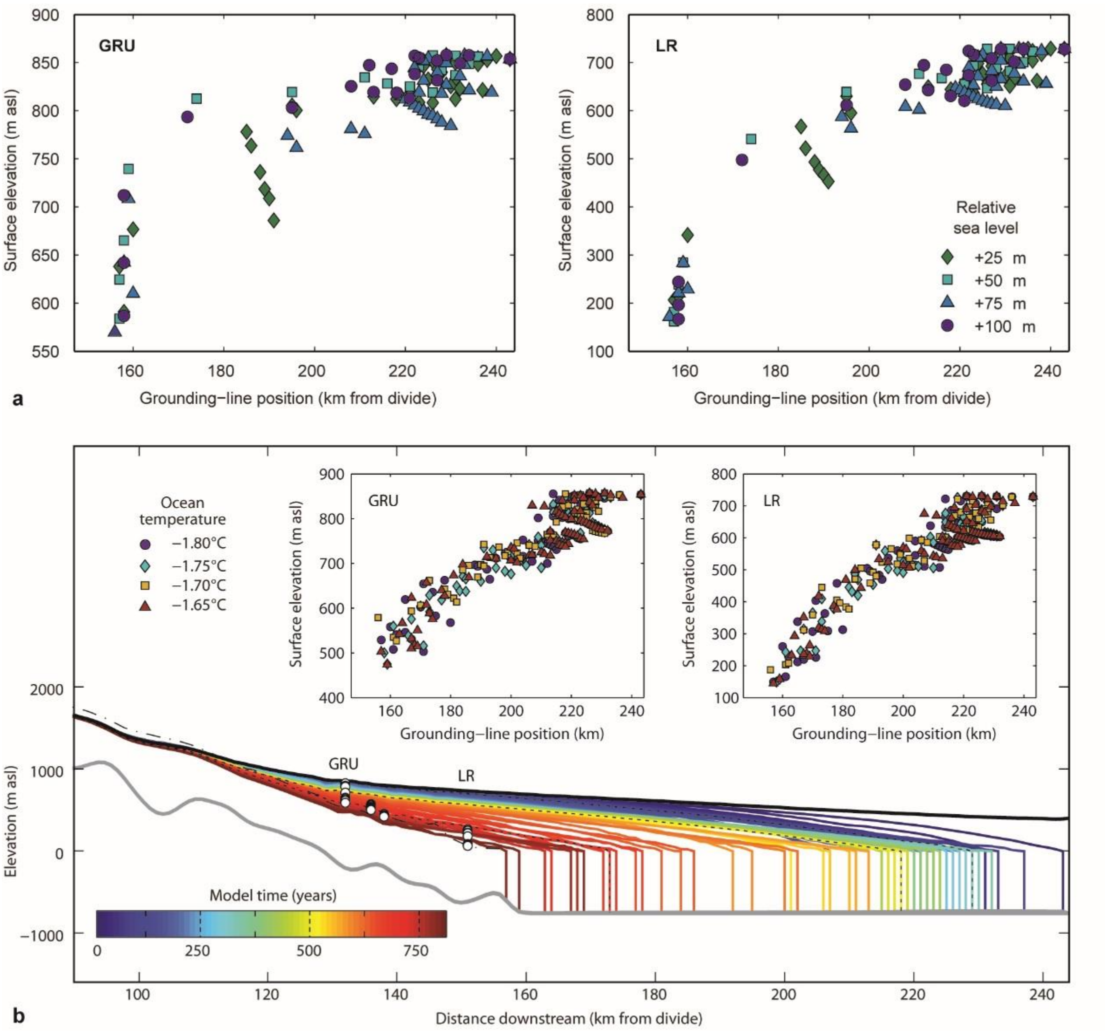

Supplementary Fig. 5. Model experiments of grounding line retreat and surface thinning at our transects. (**a**) Retreat driven by enhanced relative sea level. The pattern of surface lowering at Mt Suess/Gondola Ridge (upper) (GRU) and Low Ridge (LR), of gradual and then accelerated thinning, is largely consistent with that of enhanced ocean temperature (Fig. 4). (**b**) Simulated retreat over an imposed horizontal bed, with an evolving surface profile as in Fig. 4, shown for a -1.65 °C ocean temperature forcing scenario. Retreat lasts for ~800 years and surface lowering responds linearly. Retreat accelerates slightly towards the modern configuration, which is probably the result of a steepening surface slope and minor increases in ice flux as the grounding line approaches the high-relief Transantarctic Mountains portion. These experiments indicate that the rapid thinning recorded in our transects can be explained by a primary topographic control, largely irrespective of any single environmental forcing.

Location	Dating Context	Timing		Study
		Corr. ^{14}C yr (2 σ)	Cal. yr (2 σ)	
North of Ross Island	Retreat of grounded ice ∗	8861 ± 60	10185-10005	1
Explorers Cove	Presence of grounded ice	8340 ± 120	9522-9268	2
Scott Coast, McMurdo Sound (multiple sites)	Final unloading of grounded ice	6600 †	7520-7465	3
Cape Bird	Retreat of grounded ice	6530 ± 60	7545-7335	4
Marble Point	Ice shelf free	5480 ± 56	6420-6178	2,5
Explorers Cove	Retreat of grounded ice	5370 ± 200	6401-5916	2
Granite Harbour	Ice shelf free	5480 ± 65	6378-6242	6
Gneiss Point	Ice shelf free	5220 ± 55	6160-5906	2,5
Cape Bernacchi	Ice shelf free	4300 ± 50	4981-4846	2,5

Corrected (corr.) ^{14}C ages are derived from the original study and a recent review of regional deglaciation[7]. Calibrated (cal.) age ranges use the Marine09 calibration curve. A core sample that dates open water conditions and, by implication, minimum age of grounded ice retreat is marked with ∗. One age was derived from a relative sea level curve comprising multiple samples, therefore no uncertainty is attached (†); for calibration, we used an uncertainty of 50 years.

Supplementary Table 1. Deglaciation recorded in the vicinity of Mackay Glacier.

Parameter	Value		Unit
	Modern	Advanced	
Flow enhancement factor	8	8	-
Sliding rate factor (TAM)	5×10^{-8}	5×10^{-8}	Pa m^2 a^{-1}
Sliding rate factor (Marine)	2×10^{-5}	2×10^{-5}	Pa m^2 a^{-1}
Glen’s flow law exponent	3	3	-
Sliding exponent	2	2	-
Geothermal heat flux	58	58	mW m^{-2}
Horizontal domain resolution	1000	1000	m
Valley width	8000	8000	m
Precipitation at sea level ∗	1.5	0.4	m a^{-1}
Precipitation lapse rate ∗	-0.01	-0.01	m a^{-1} km^{-1}
Mean annual temp. at sea level †	-17	-17	°C
Temperature lapse rate	-9	-9	K km^{-1}
Annual temperature range †	35	35	°C
Ocean temperature (winter)	-1.8	-1.8	°C
Ocean temperature (summer) ∗	-1.6	-1.8	°C
Calving rate coefficient ∗	1	0.3	-
Relative sea level	0	0	m
Degree-day factor	0.005	0.005	mm K^{-1} d^{-1}

Modern climate parameters were obtained from National Soil Survey Centre / Landcare Research (marked with †). Less certain parameters were tuned to simulate the desired glacier profile (∗). Reduced precipitation is assumed for the advanced profile based on ice core interpretation[8]. Sliding rate factors are stated for the Transantarctic Mountains (TAM) and marine portions.

Supplementary Table 2. Flowline model parameters.

Supplementary Note 1

Multiple onshore and offshore records constrain the timing and extent of past ice retreat in the western Ross Sea (Supplementary Table 1). For comparison between this regional evidence and our new glacier thinning transects, all ^{14}C dates have been corrected for marine reservoir effects and calibrated using the marine calibration curve (Marine09) as recommended by their original sources and a recent review of regional deglaciation[7]. Moreover, dates obtained from offshore cores were only included if they showed linear sedimentation rates and consistent ^{13}C values through the core[6]. Immediately north of Ross Island, a minimum age for grounding line and ice shelf retreat is recorded at 10,185–10,005 calibrated years BP, based on its core-top reservoir correction of 2,470 years[1]. However, deglaciation may have occurred slightly earlier in this location if a smaller, regional, reservoir correction (e.g. 1,200–1,300 years) is used instead.

Local evidence of Mackay Glacier retreat is also apparent offshore as a series of grounding-zone wedges (GZWs) preserved in the outer part of the trough (Supplementary Fig. 4). These suggest that the initial retreat of the grounding line was most likely staggered. Although we do not have data to constrain the timing and duration of these stillstands, studies of similar-sized GZWs in West Antarctica indicate that they typically form within ~120 years if the sediment flux is sufficiently high[13].

Supplementary References

1 McKay, R. *et al.* Retreat history of the Ross Ice Sheet (Shelf) since the Last Glacial Maximum from deep-basin sediment cores around Ross Island. *Palaeogeography, Palaeoclimatology, Palaeoecology* **260**, 245-261 (2008).

2 Hall, B. L. & Denton, G. H. Extent and chronology of the Ross Sea ice sheet and the Wilson Piedmont Glacier along the Scott Coast at and since the last glacial maximum. *Geografiska Annaler: Series A, Physical Geography* **82**, 337-363 (2000).

3 Hall, B. L., Baroni, C. & Denton, G. H. Holocene relative sea-level history of the Southern Victoria Land Coast, Antarctica. *Global and Planetary Change* **42**, 241-263 (2004).

4 Licht, K. J., Jennings, A. E., Andrews, J. T. & Williams, K. M. Chronology of late Wisconsin ice retreat from the western Ross Sea, Antarctica. *Geology* **24**, 223-226 (1996).

5 Hall, B. L. & Denton, G. H. New relative sea-level curves for the southern Scott Coast, Antarctica: evidence for Holocene deglaciation of the western Ross Sea. *Journal of Quaternary Science* **14**, 641-650 (1999).

6 Domack, E. W., Jacobson, E. A., Shipp, S. & Anderson, J. B. Late Pleistocene–Holocene retreat of the West Antarctic Ice-Sheet system in the Ross Sea: Part 2—sedimentologic and stratigraphic signature. *Geological Society of America Bulletin* **111**, 1517-1536 (1999).

7 Anderson, J. B. *et al.* Ross Sea paleo-ice sheet drainage and deglacial history during and since the LGM. *Quaternary Science Reviews* **100**, 31-54 (2014).

8 Steig, E. J. *et al.* Wisconsinan and Holocene climate history from an ice core at Taylor Dome, western Ross Embayment, Antarctica. *Geografiska Annaler: Series A, Physical Geography* **82**, 213-235 (2000).

9 Balco, G., Stone, J. O., Lifton, N. A. & Dunai, T. J. A complete and easily accessible means of calculating surface exposure ages or erosion rates from ^{10}Be and ^{26}Al measurements. *Quaternary Geochronology* **3**, 174-195 (2008).

10 Putnam, A. *et al.* In situ cosmogenic ^{10}Be production-rate calibration from the Southern Alps, New Zealand. *Quaternary Geochronology* **5**, 392-409 (2010).

11 Greenwood, S. L., Gyllencreutz, R., Jakobsson, M. & Anderson, J. B. Ice-flow switching and East/West Antarctic Ice Sheet roles in glaciation of the western Ross Sea. *Geological Society of America Bulletin* **124**, 1736-1749 (2012).

12 Calkin, P. E. Subglacial geomorphology surrounding the ice-free valleys of southern Victoria Land, Antarctica. *Journal of Glaciology* **13**, 415-429 (1974).

13 Graham, A. G. *et al.* Flow and retreat of the Late Quaternary Pine Island-Thwaites palaeo-ice stream, West Antarctica. *Journal of Geophysical Research: Earth Surface (2003–2012)* **115**, doi: 10.1029/2009JF001482 (2010).

14 Lal, D. Cosmic ray labeling of erosion surfaces: *in situ* nuclide production rates and erosion models. *Earth and Planetary Science Letters* **104**, 424-439 (1991).

15 Stone, J. O. Air pressure and cosmogenic isotope production. *Journal of Geophysical Research: Solid Earth (1978–2012)* **105**, 23753-23759 (2000).

16 Desilets, D. & Zreda, M. Spatial and temporal distribution of secondary cosmic-ray nucleon intensities and applications to in situ cosmogenic dating. *Earth and Planetary Science Letters* **206**, 21-42 (2003).

17 Dunai, T. J. Scaling factors for production rates of in situ produced cosmogenic nuclides: a critical reevaluation. *Earth and Planetary Science Letters* **176**, 157-169 (2000).

18 Lifton, N. A. *et al.* Addressing solar modulation and long-term uncertainties in scaling secondary cosmic rays for in situ cosmogenic nuclide applications. *Earth and Planetary Science Letters* **239**, 140-161 (2005).

19 Nishiizumi, K. *et al.* Cosmic ray production rates of ^{10}Be and ^{26}Al in quartz from glacially polished rocks. *Journal of Geophysical Research: Solid Earth (1978–2012)* **94**, 17907-17915 (1989).
